# Multimodal 3D photoacoustic remote sensing and confocal fluorescence microscopy imaging

**DOI:** 10.1117/1.JBO.26.9.096501

**Published:** 2021-09-15

**Authors:** Brendon S. Restall, Pradyumna Kedarisetti, Nathaniel J. M. Haven, Matthew T. Martell, Roger J. Zemp

**Affiliations:** University of Alberta, Department of Electrical and Computer Engineering, Edmonton, Canada

**Keywords:** optics, photoacoustic, microscopy, fluorescence, confocal, 3D, absorption-contrast, cell nuclei

## Abstract

**Significance:** Complementary absorption and fluorescence contrast could prove useful for a wide range of biomedical applications. However, current absorption-based photoacoustic microscopy systems require the ultrasound transducers to physically touch the samples, thereby increasing contamination and limiting strong optical focusing in reflection mode.

**Aim**: We sought to develop an all-optical system for imaging cells and tissues using the three combined imaging modalities: photoacoustic remote sensing (PARS), epifluorescence, and confocal laser scanning microscopy (CLSM).

**Approach:** A PARS subsystem with ultraviolet excitation was used to obtain label-free absorption-contrast images of nucleic acids in *ex vivo* tissue samples. Co-integrated epifluorescence and CLSM subsystems were used to verify the 2D and 3D nuclei distribution.

**Results**: Complementary absorption and fluorescence contrast were demonstrated in phantom imaging experiments and subsequent cell and tissue imaging experiments. Lateral and axial resolution of ultraviolet-PARS (UV-PARS) is shown to be 0.39 and 1.6  μm, respectively, with 266-nm light. CLSM lateral and axial resolution was measured as 0.97 and 2.0  μm, respectively. This resolution is sufficient to image individual cell layers with fine optical sectioning. UV-PARS images of cell nuclei are validated in thick tissue using CLSM.

**Conclusions**: Multimodal absorption and fluorescence contrast are obtained with a non-contact all-optical microscopy system for the first time and utilized to obtain images of cells and tissues with subcellular resolution.

## 1 Introduction

Fluorescence microscopy is a proven technology that has enabled the previously unattainable visualization of a multitude of molecular targets in samples. Many exogenous and genetically encoded fluorescent probes have been developed that have revolutionized biomedicine.^1^^–^^5^ However, fluorescence microscopy also has limitations. Typically, due to the reliance on exogenous fluorescence labels, the labeling of multiple target molecules can be complicated due to emission spectra overlap thereby limiting imaging to four or five fluorescent markers.^6^^–^^8^

Imaging with both absorption and fluorescence contrast could enable important applications in cell biology such as intravital imaging of model organisms and tissues. Photoacoustic microscopy (PAM) is an emerging modality that offers optical absorption contrast. Previous work using optical resolution photoacoustic microscopy (OR-PAM) has demonstrated the ability to image vasculature, cytochromes, and cell nuclei by tuning the excitation laser pulses to specific wavelengths corresponding to absorption peaks of the desired chromophores.^9^^,^^10^ When combined with fluorescence, micrometastases and cancerous regions labeled with tumor markers have enabled the study of the tumor microenvironment and the assessment of antineoplastic drugs.^11^^–^^16^ However, most current PAM systems are not yet all optical and require ultrasound transducers. These transducers require acoustic coupling and limit strong optical focusing in reflection mode.

To circumvent these issues, we have developed an all-optical system providing fluorescence microscopy combined with absorption-contrast photoacoustic remote sensing (PARS) microscopy. Both imaging modalities demonstrate high sensitivity and specificity.^17^^–^^19^ PARS does not require an ultrasound transducer, acoustic coupling, or physical contact with the specimen. This modality instead relies on detecting reflected modulations of an interrogation beam due to the initial photoacoustic pressures generated by the absorption of a pulsed excitation beam by the chromophores of interest in selective tissues such as breast, gastrointestinal, and lung.^20^^–^^22^ Multicontrast imaging has recently been demonstrated, where multiple pulsed excitation wavelengths are interlaced to generate absorption contrast from several chromophores.^23^^–^^26^ In this paper, we demonstrate a combined contrast imaging system for phantom imaging experiments and further apply the system to cell and tissue imaging applications and to validate the label-free 3D virtual histology capabilities of ultraviolet-PARS (UV-PARS) using fluorescent labeling and confocal microscopy. This is the first report combining optical absorption and confocal fluorescence contrast in a high-resolution, non-contact, optical imaging system. To date, one missing comparison is that UV-PARS histology has previously demonstrated absorption-contrast imaging of cell nuclei but has yet to be validated in thick tissue sections.^20^^,^^27^^,^^28^ The validation of the 3D virtual histology capabilities of UV-PARS is an important step toward label-free tissue imaging both *ex vivo* and *in vivo.* The combination of confocal laser scanning microscopy (CLSM) and the absorption contrast gained with UV-PARS could help augment intraoperative surgery by spot checking margin status in areas of interest in fluorescence guided surgeries as well as replace the hematoxylin and eosin (H&E) staining histological analysis post-surgery with a much faster and less labor-intensive imaging technique that could be employed intraoperatively. When paired with fluorescence guided surgery, our technology could both visualize fluorescent agents intended to label tumors or nerves as well as provide histological details. More broadly, the combined absorption and fluorescence contrast afforded by the presented system could enable future studies involving an expanded palette of endogenous and exogenous agents for biological and medical imaging applications.

## 2 Methods

### 2.1 Experimental Setup

**Fig. 1:**
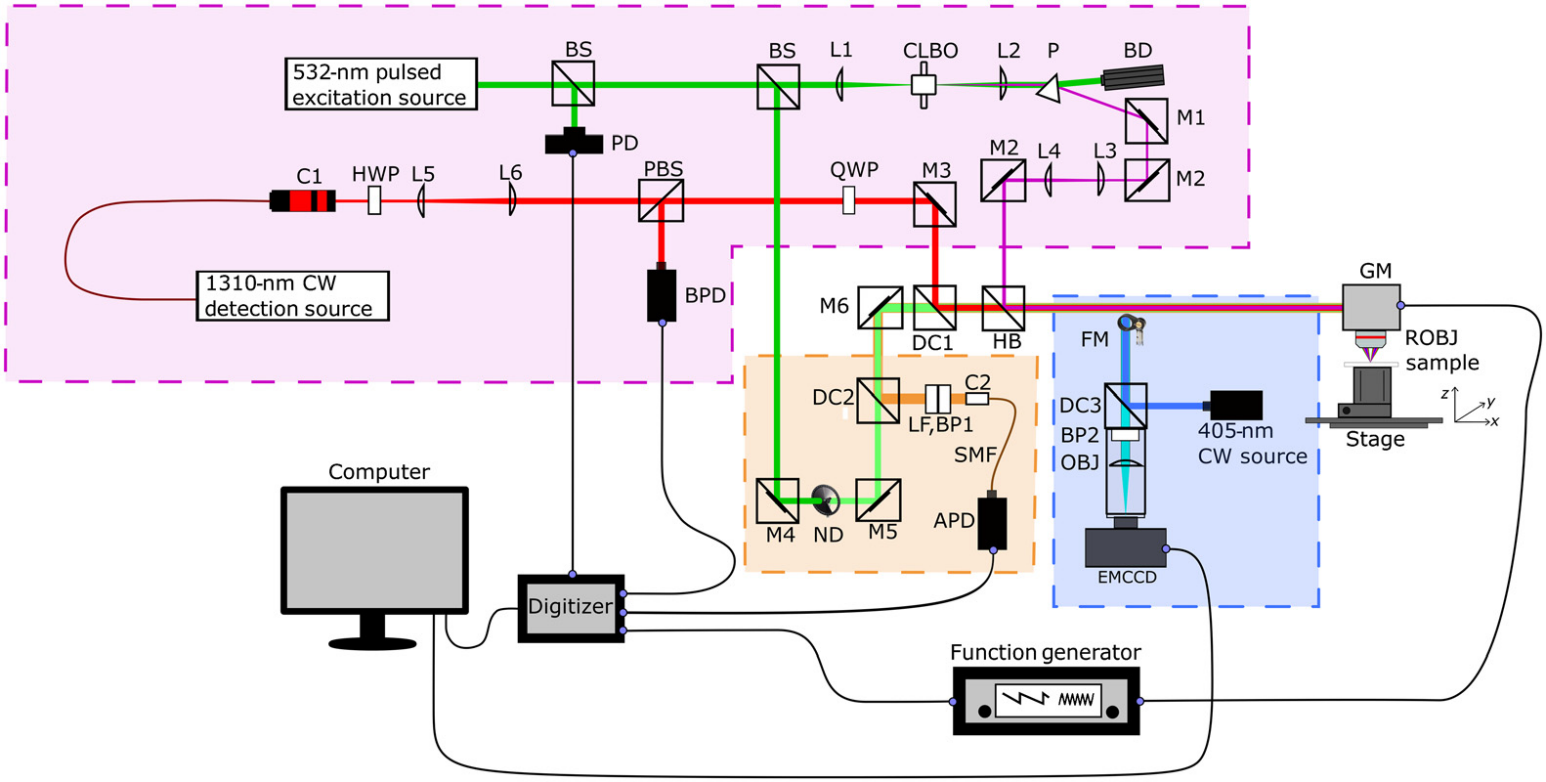
System diagram of UV-PARS (shaded purple), camera-based epifluorescence system (shaded blue) and laser scanning confocal fluorescence microscopy (shaded orange). Components can be identified as: avalanche photo-diode (APD), balanced photodiode (BPD), bandpass filter (BP), beam dump (BD), beamsplitter (BS), caesium lithium borate (CLBO) crystal, collimator (C), dichroic mirror (DC), electron multiplied charge coupled device (EMCCD) camera, flip mirror (FM), galvanometer mirrors (GM), half-wave plate (HWP), harmonic beam splitter (HB), laser line filter (LF), mirror (M), neutral density filter (ND), objective lens (OBJ), photodiode (PD), polarized beam splitter (PBS), prism (P), quarter-wave plate (QWP), and reflective objective (ROBJ).

The experimental setup is comprised of three subsystems as shown in Fig. 1: the UV-PARS subsystem, a camera-based epifluorescence subsystem, and a CLSM subsystem.

The UV-PARS subsystem is similar to that reported by Haven et al. (2019).^28^ To briefly summarize, a 1310-nm continuous-wave interrogation beam (Thorlabs, SLD1018PXL) is co-aligned with a 266-nm pulsed excitation beam, generated by frequency doubling the output of a 40-kHz 532-nm nanosecond pulsed fiber laser (IPG Photonics, GLP-10) using a caesium lithium borate (CLBO) (Eksma Optics) crystal. These two beams are then directed through a galvanometer scanning mirror system then a 0.5 NA dry reflective objective (Thorlabs, LMM-40X-UVV). A pulse energy of 5 nJ of 266-nm light and 12.5 mW of 1310-nm light is focused onto the sample. Both beams are expanded to maximally fill the aperture of the reflective objective when scanned through it. The back-reflections of these beams are separated using a harmonic beam splitter (DC1, Thorlabs, HBSY134) through which the 266-nm back-reflection signal is discarded, and the 1310-nm backscatter signal is detected by a 75-MHz balanced photodiode (Thorlabs, PDB420C-AC).

This work employed the use of two fluorophores, propidium iodide (PI), and proflavine, to label cell nuclei. Both stains intercalate between nucleotides in both DNA and RNA. PI’s excitation peak is situated at 535 nm and emission peak at 617 nm,^29^ and proflavine has an excitation peak at 460 nm and emission peak at 515 nm.^30^ PI was selected for its ease of integration with the UV-PARS system. We selected the fluorescence stain that would be excited at 532 nm and exhibit a large red-shift emission, which would bind to cell nuclei. We chose proflavine as a fluorescent marker as it did not induce significant inference with UV-PARS in our experiments and has been used in the previous literature as a marker of cell nuclei.^30^^–^^32^

Epifluorescence imaging was realized using an electron multiplied charge coupled device (EMCCD) camera (Andor, DV-885K-CSO-#VP) to capture images. To acquire epifluorescence images prior to UV-PARS imaging, a flip mirror was used to redirect the optical path of the system toward the epifluorescence system. A 405-nm continuous-wave excitation laser (Thorlabs, CPS405) is used as the fluorescence excitation source for proflavine and fed into the reflective objective with 2.0 mW illuminating the sample. The generated fluorescence from the sample is collimated back through the objective and is isolated from the excitation light using a 480-nm cut-off long pass dichroic (DC3, Thorlabs, MD480) and is further band passed through a 520-nm/14-nm filter (BP2, Thorlabs, MDF-GFP2). This fluorescence emission is then focused onto the EMCCD using an objective lens (Thorlabs, LSM02-VIS) to maximally fill the EMCCD aperture.

The addition of a confocal fluorescence subsystem utilized 532-nm excitation light and an avalanche photodiode while operating simultaneously with UV-PARS imaging. A 70:30 beamsplitter (Thorlabs, BST10) was used to split the 532-nm excitation from the main optical pathway for UV generation with the 30% pick-off beam being used to excite the fluorophore, PI. The CLSM system utilized a 605-nm cut-off long-pass dichroic filter (DC2, Thorlabs, DMLP605R) and a 630-nm/69-nm bandpass filter (BP1, Semrock, FF01-630/69-25). The intensity of fluorescence excitation was controlled using a continuous ND filter wheel (Thorlabs, NDC-50C-4M) to lower the pulse energy on the sample to 45 nJ. The 532-nm light was co-aligned back into the main optical pathway with the UV excitation and 1310-nm interrogation beam and point scanned across the sample through the reflective objective. The fluorescence emission traveled back through the reflective objective and was redirected toward a detection pathway using a long pass 550-nm dichroic filter (Thorlabs, DMLP550R). To further remove any other unwanted wavelengths of light, the fluorescence emission was filtered using a 532-nm laser notch filter (Thorlabs, NF533-17) and 630-nm/69-nm bandpass filter set (Thorlabs, MF630-69). The fluorescence emission was then collimated (Thorlabs, F280FC-A) and fed into a single-mode fiber (SMF) (Thorlabs, P5-460B-PCAPC-1) with a mode field diameter range of 2.8 to 4.1  μm. The SMF effectively functions as a pinhole for spatial filtering to achieve the desired confocal sectioning comparable to UV-PARS, with the output end of the fiber directly coupled to an avalanche photodiode (Thorlabs, APD120A2).

For digital data acquisition, we utilized four channels of a 12-bit, 125  MS/s data acquisition card (GaGe, CSE8389-2GS). The first channel was used to acquire the UV-PARS signal from the RF output of the balanced photodiode, the second and third channels were used to record the fast axis and slow axis position of a galvanometer scanning system, whereas the fourth channel was used to acquire fluorescence data from the avalanche photodiode. The RF signal from the balanced photodiode was bandpass filtered between 1.8 to 22 MHz to remove fluctuations due to scanning and reject electronic noise to form the basis for our detected UV-PARS signal. Acquisition triggering was controlled using the output of a photodiode, which monitored a low-power pick-off from the 532-nm excitation source.

Data for all four channels were collected with 32 samples recorded post trigger. All digitized data was processed in MATLAB. For each trigger, data was averaged for the galvanometer and fluorescence signals while the Hilbert transform was used to obtain the rectified envelope of the UV-PARS waveform; the maximum value of which is used as the UV-PARS signal intensity. A total of 200,000 data points were captured for each image that corresponded to an average physical spacing of 336 nm between pulses on the 150  μm×150  μm sample field of view (FOV). The laser was scanned across the sample for each tile using the galvanometer then translated using an axial stage (Zaber, X-VSR-E) and X−Y motorized stage (Thorlabs, MLS203-1). The separate galvanometer images were stitched together in MATLAB using the X−Y mechanical stage positional data to crop the overlap between each FOV. We used a 40-kHz pulse-repetition rate and acquired 200,000 points per image with galvanometer slow and fast axis frequencies of 0.20 and 63.2 Hz, respectively, to ensure equal spacing between each interrogation point on the sample. With these parameters, an image acquisition time of 5 s per 150  μm×150  μm FOV was achieved.

### 2.2 Sample Preparation

All animal samples were acquired in accordance with the University of Alberta’s Animal Care and Use Committee ethics guidelines and regulations. Organ tissue sections were dissected from a nude mouse (Charles River, NU/NU) following euthanasia. Dissected tissues were washed with phosphate buffered saline and immersed in 10% neutral buffered formalin for fixation, embedded in paraffin blocks, and finally sectioned to the desired thickness of 4 or 30  μm and adhered to a glass slide. Prior to imaging, deparaffination and rehydration were performed by first heating the slides at 60°C for 1 h, followed by 2-min-long washes in 2 changes of xylene, 2 changes of 100% ethanol, 95% ethanol, and finally deionized water (DI). For fluorescence imaging, samples were then washed three times with phosphate buffered saline and incubated in 100  μg/mL RNase (ThermoFisher, EN0531) for 60 min at room temperature to remove RNA from the cell. The samples were then equilibrated in phosphate buffered saline, stained with 300  μL of 500 nM PI (ThermoFisher, P1304MP), and incubated at room temperature for 5 min. Finally, the sample was rinsed three times with phosphate buffered saline prior to imaging. While imaging was taking place, the sample was covered with a UV coverslip and DI water.

### 2.3 Cell Culture Preparation

HeLa cells (ATCC, CCL-2), a model cancer cell line, were imaged in this study. These cells were subcultured and grown onto fibrinogen-coated glass coverslips in Dulbecco’s Modified Eagle Medium supplemented with 10% fetal bovine serum and 1% penicillin–streptomycin. Once cell cultures grew to ∼50% confluency, the coverslips were washed three times in phosphate buffered saline and fixed by incubation in 4% paraformaldehyde for 30 min at 37°C. The cells were then washed three times with DI water and then stored for subsequent UV-PARS and/or fluorescence microscopy imaging experiments.

For nuclear staining of these cell cultures, the coverslips were gently washed three times with PBS and incubated in 100  μg/mL RNase for 60 min at room temperature to remove RNA from the cells. The coverslips were then incubated in 0.01% w/v proflavine (Sigma Aldrich, P2508-10G) at room temperature for 10 min. Then the coverslips were washed with DI water three times. The RNA removal step prior to fluorescent labeling aided in increasing nuclear specificity and prevented sequestration of the fluorophores by cytoplasmic RNA.

## 3 Results

### 3.1 Resolution Characterization

**Fig. 2:**
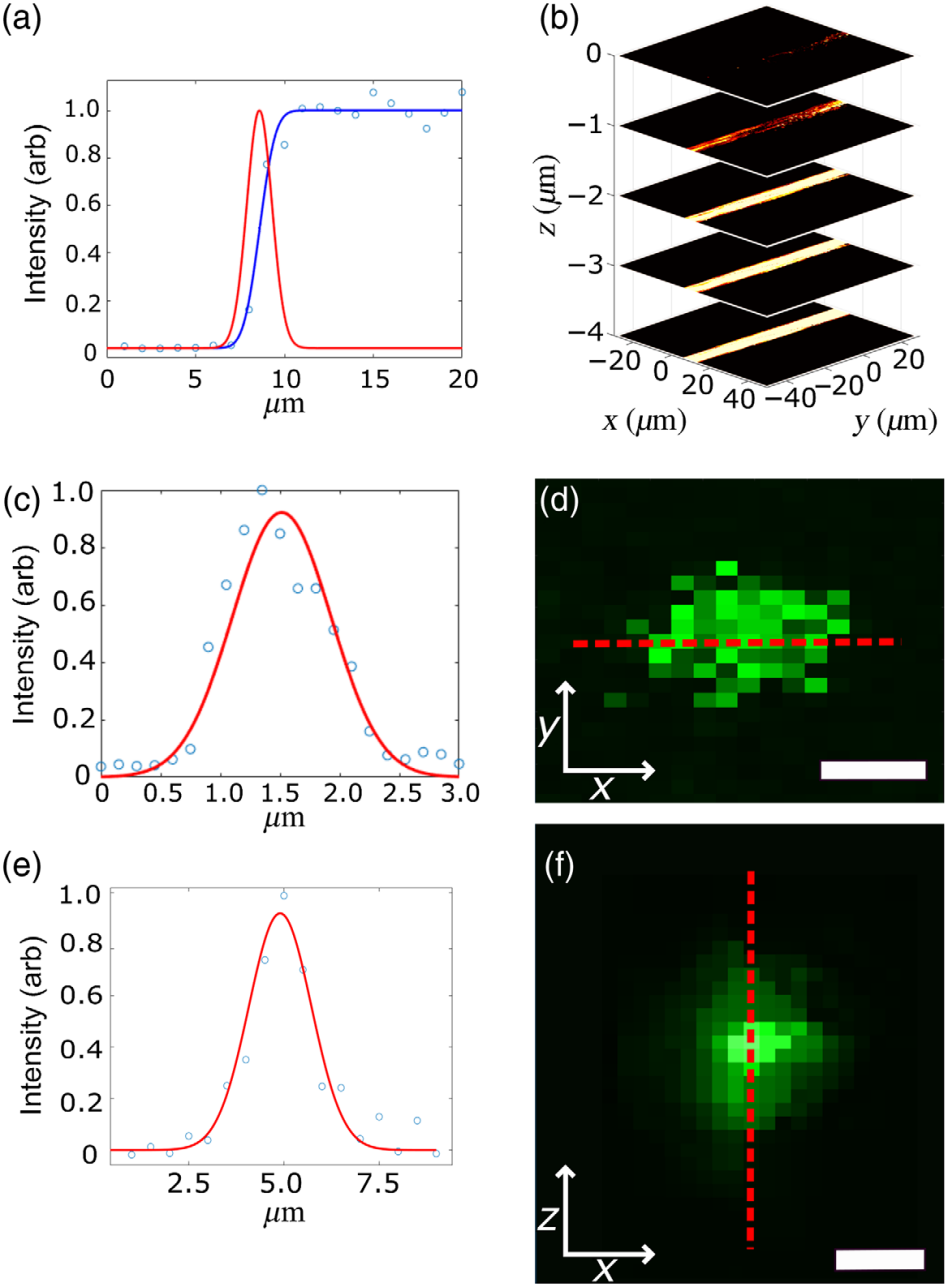
Resolution characterization. (a) The ESF of a carbon fiber using UV-PARS taken with sequential axial images. Images were taken at 1  μm intervals from the top of the fiber downward. (b) Topographical view of each fiber images as the sample was raised to optically section it at 1  μm intervals. (c) Gaussian fit intensity plot of a 0.1-μm diameter fluorescent microbead. The FWHM is used to calculate the lateral resolution. (d) Fluorescence image of the fluorescent microbead and the line profile used for the intensity plot. Scale bar is 1  μm. (e) Axial Gaussian fit of intensity plot for a single 0.5  μm fluorescent microbead. (f) Axial image z-stack of fluorescent microbead taken at 0.5  μm intervals, then projected along the y axis. Scale bar is 1  μm.

Lateral resolution of our UV-PARS subsystem was measured by imaging 100-nm gold nanospheres (Sigma-Aldrich, 742031). This lateral resolution has been characterized to be 0.39  μm by measuring the smallest resolved distance between two adjacent nanoparticles with a distinguishable full-width at half-maximum (FWHM) as shown in the previous work.^28^ The optical sectioning capabilities and axial resolution of the UV-PARS subsystem were characterized by acquiring the intensity profile of a carbon fiber with incremental steps of 1  μm along a z-stack. An edge spread function (ESF) was plotted from the intensity profile of the carbon fiber and subsequently the line spread function (LSF) was calculated using the derivative of the ESF. A Gaussian profile was fit to the LSF characterization, which resulted in a FWHM axial resolution of 1.6  μm [Figs. 2(a) and 2(b)].

To characterize the lateral resolution of the CLSM subsystem, 0.1-μm diameter fluorescent microspheres (Thermofisher, T14792) were imaged using the 532-nm excitation source. A Delaunay triangulation algorithm was implemented to create a discrete 2D pixel image from our scattered data. The lateral resolution in fluorescence images was calculated using a Gaussian fit of the nanoparticles’ FWHM intensity profile where the lateral resolution was determined to be 0.97  μm [Figs. 2(c) and 2(d)]. This is twice as large as the theoretical value of 0.46  μm, calculated for a confocal microscope using a 0.5-NA objective at the peak emission wavelength of 617 nm for PI as per Eq. 8.34 of Wang and Wu 2007^33^. The discrepancy between the theoretical and our measured values for lateral resolution is likely due to underfilling of the reflective objective aperture, which was intentionally not maximized in order to provide sufficient room for the galvanometer scanning.

Axial resolution of the CLSM subsystem was determined by acquiring 18 images translating in the z direction to create a 3D z-stack. We then took the average intensity of each y column vector for each of the z plane images to create a 2D z stack of the fluorescence microsphere. Finally, we took the FWHM of the intensity line plot across the region of interest containing the microsphere. A Gaussian plot was fit to the intensity profile with the FWHM being used to calculate an axial resolution of 2.0  μm [Figs. 2(e) and 2(f)]. For comparison, a theoretical value of 1.84  μm was calculated using the axial PSF for a confocal microscope at the peak emission wavelength for PI at 617 nm as per Eq. 8.31 of Ref. 33.

**Fig. 3:**
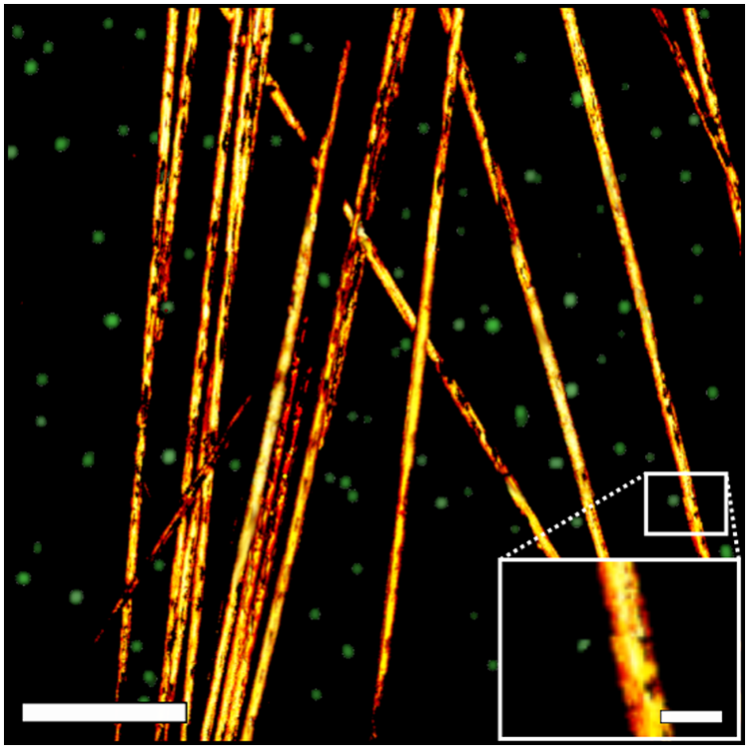
Carbon fiber absorption contrast and fluorescence microbead fluorescence emission phantom image. Carbon fibers are shown in the foreground and the enlarged microbeads are in the background. Scale bar is 100  μm. See inset in the lower right-hand corner for actual size of microbeads compared to the carbon fibers. Scale bar is 10  μm.

To demonstrate complementary absorption and fluorescence contrast imaging with our multimodal system, we imaged phantoms consisting of both 7  μm carbon fibers (for absorption contrast) and 0.5  μm fluorescent microbeads (Invitrogen, T7281). Fluorescence emission is captured with the CLSM subsystem simultaneously with the UV-PARS data in a 3×3 mosaic of 155  μm×155  μm scans, as shown in Fig. 3. The microbeads have been enlarged via a dilation image processing operation to allow the viewer to observe the two imaging modalities superimposed upon each other. Without this the step, the microbeads were poorly visible in the image due to the size differences between the carbon fibers and microbead phantoms. The images are acquired by point scanning across the sample using our two-axis galvanometer scanning system for each section of the mosaic then translating the sample using our x−y stage for the next section.

### 3.2 Epifluorescence and UV-PARS

After resolution characterization and complimentary multimodal capabilities, we demonstrate the utility of our system by imaging cells and tissues. Fluorescence images were first obtained via the epifluorescence path using the EMCCD camera to obtain static fluorescent images prior to subsequent UV-PARS images. A flip mirror was moved into place before the epifluorescence image was taken. We observed the performance of UV-PARS for imaging cell nuclei compared to epifluorescence imaging with cell cultures and both thin and thick tissue sections where we identified morphological similarities between the two imaging modalities. However, the inherent inability of epifluorescence to image thick tissue and achieve optical sectioning posed a serious problem. As expected, out of focus fluorescence is not rejected in thick sections with epifluorescence, thereby reducing contrast and producing artifacts from cells and tissues outside the desired focal plane. Epifluorescence was used to demonstrate the ease of access to add an additional, albeit asynchronous, imaging modality to the existing UV-PARS system as well as demonstrate the improvements that could be provided by the more complex CLSM subsystem. Epifluorescence can be considered a viable subsystem to add to UV-PARS as long as there is not a great need for simultaneous absorption and fluorescence contrast or single-slice imaging in thick tissue.

**Fig. 4:**
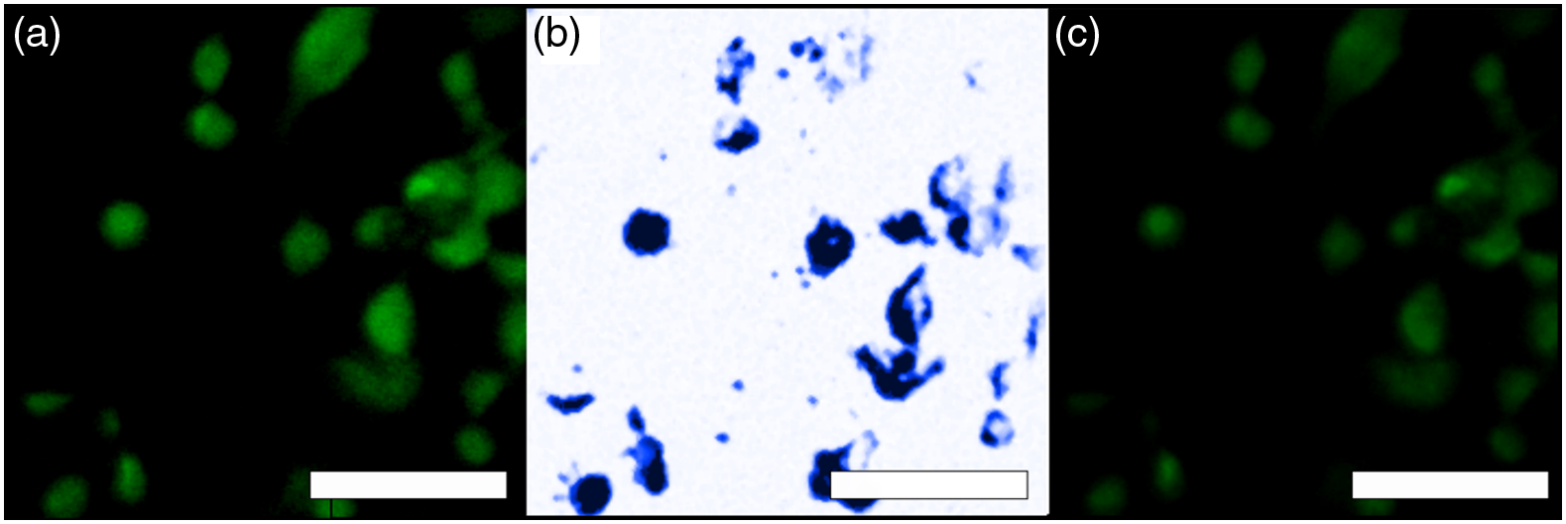
Fixed HeLa cells are shown with both absorption contrast and fluorescence contrast using proflavine. (a) Fluorescence image taken before UV-PARS image was acquired; (b) UV-PARS image; and (c) fluorescence image taken after UV-PARS image was acquired. Scale bars are all 50  μm.

Imaging of HeLa cell cultures was used to compare the correlation between cell nuclei images obtained with camera epifluorescence of PI and UV-PARS (Fig. 4). FOVs containing up to 10 cells each were imaged to provide two images for comparison of their intensity alignment using colocalization in ImageJ. The images of fixed HeLa cells had excellent correlation but with a varying intensity between the two imaging modalities. Colocalization of fluorescence and UV-PARS signals was analyzed using Li’s intensity correlation analysis (ICA).^34^ Li’s intensity correlation quotient was measured as a 0.44 out of a maximum of 0.5, suggesting a very high degree of colocalization. Fluorescence images of cells were taken before [Fig. 4(a)] and after [Fig. 4(c)] UV-PARS imaging [Fig. 4(b)]. Signal normalization was maintained between the two fluorescence images to allow for an accurate portrayal of the change in signal between images. A decrease in fluorescence signal intensity of ∼8.9% was observed after UV-PARS imaging. Comparatively, in two confocal images taken sequentially, a 1.3% decrease in fluorescence signal intensity was observed. This demonstrates that there is a small photobleaching effect as the nuclei are not as prominent post-UV-PARS exposure, but also that there is no excessive damage to the sample itself as the power exposures of 5 and 45 nJ for the 266- and 532-nm light, respectively, are below the American National Standards Institute limits.^23^

**Fig. 5:**
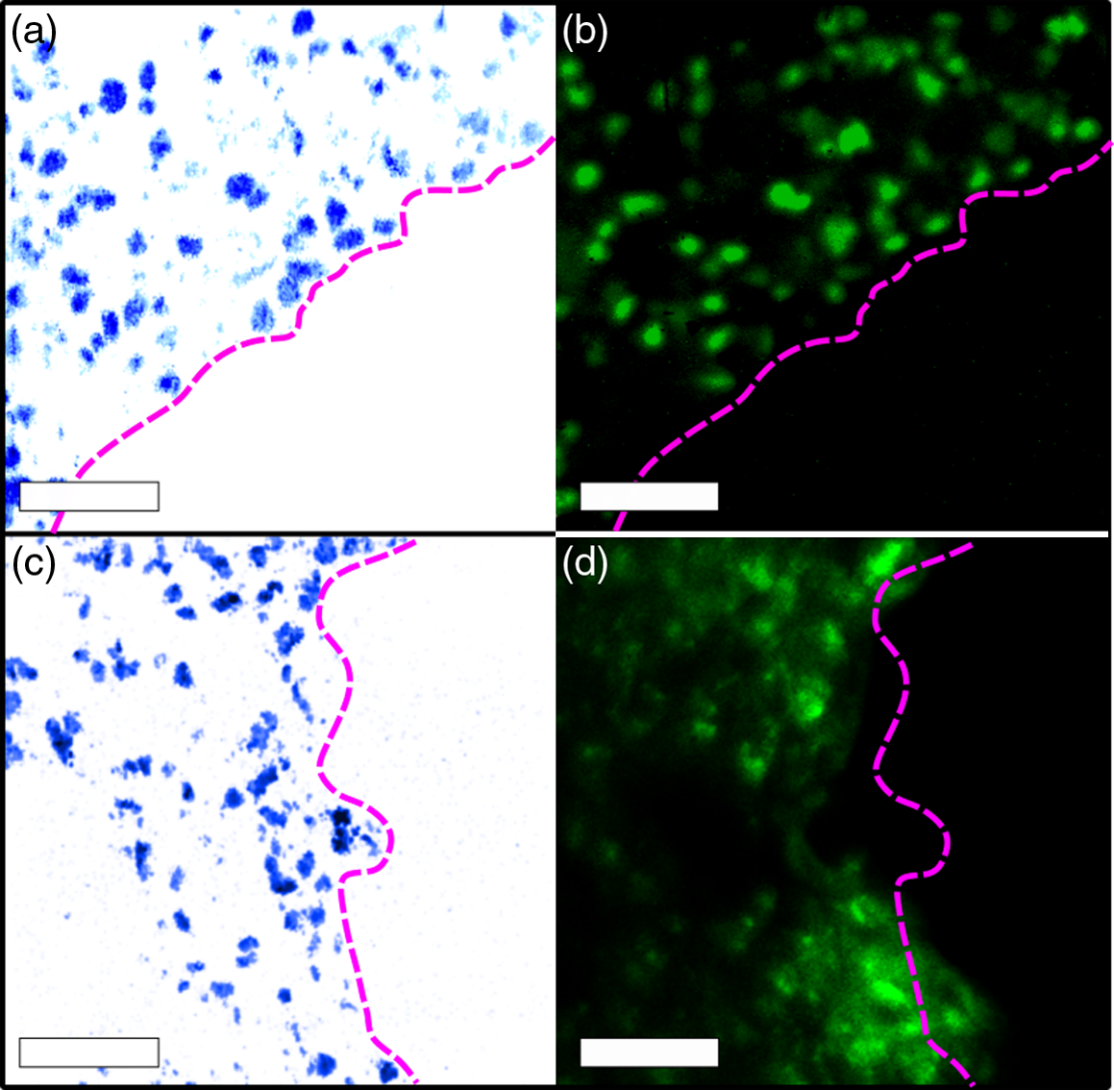
(a) UV-PARS of 4  μm sectioned mouse kidney; (b) camera fluorescence images of 4  μm mouse kidney stained with proflavine; (c) UV-PARS of 30  μm sectioned mouse lung; and (d) camera fluorescence images of 30  μm mouse lung stained with proflavine. Scale bars are all 50  μm.

Imaging of kidney tissue stained with proflavine using epifluorescence microscopy produced comparisons for UV-PARS in both thick and thin tissue sections. The borders of the tissue were marked for both UV-PARS [Fig. 5(a)] and fluorescence [Fig. 5(b)] imaging showing observable similarities between many identifiable features. This demonstrates a strong correlation between images of the fluorescently stained cell nuclei and UV-PARS absorption in thin tissue sections with a Li’s ICA value of 0.317. These results are promising for the purpose of validation of UV-PARS, however, epifluorescence microscopy with thick tissue sectioning poses a critical issue of being unable to optically section individual cell layers. UV-PARS has been demonstrated to achieve optical sectioning on the order of 1.6  μm when using tight optical focusing, allowing for single-cell layer imaging of cell nuclei in thick tissues. Images with UV-PARS [Fig. 5(c)] and epifluorescence [Fig. 5(d)] show low colocalization due to the larger imaging depth-of-focus in the epifluorescence image. Still, there are some observable similarities, and the correlation was quantified as a moderate value of 0.155 using Li’s ICA. The fluorescence image appears blurry due to some uptake of proflavine in the cell cytoplasm as well as multiple layers of cell nuclei in the thicker tissue.

### 3.3 Confocal Laser Scanning Microscopy and UV-PARS

To overcome the large depth of focus encountered with epifluorescence, CLSM was demonstrated and compared in the same system to UV-PARS for thicker samples obtaining layer by layer images of cell nuclei. This system operates in conjunction with the existing UV-PARS system to enable faster data acquisition, additional contrast using both absorbance and fluorescent labels, and point by point comparisons between images taken simultaneously.

**Fig. 6:**
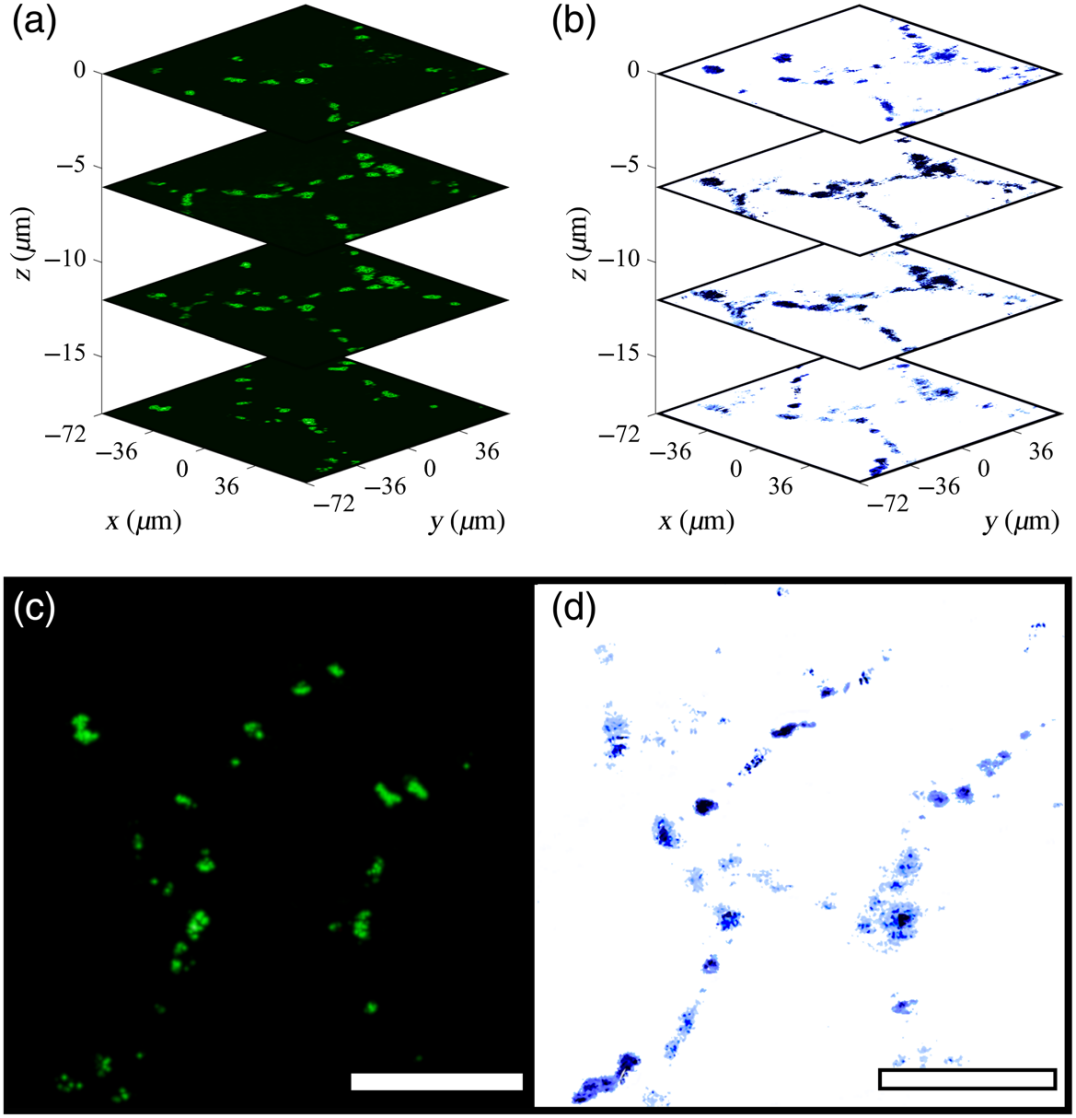
3D imaging of a thick tissue section of a mouse lung. Images were sectioned at 6  μm intervals. (a) CLSM imaging of cell nuclei stained with PI; (b) UV-PARS absorption contrast of cell nuclei; (c) CLSM slice at the maximum depth of 24  μm; and (d) UV-PARS slice of absorption contrast at maximum imaging depth. Scale bars are 50  μm.

Three dimensional UV-PARS was validated with CLSM in 30-μm thick paraffin embedded mouse lung tissue. Lung tissue was imaged at four different depths starting from 6  μm below the surface then at 6  μm intervals with both CLSM [Fig. 6(a)] and UV-PARS [Fig. 6(b)] to image the cell nuclei. Both imaging modalities are able to provide nuclear contrast at each depth with an average Li’s ICA value of 0.445±0.013 for the four images. Traditional validation using the hematoxylin staining of nuclei and brightfield imaging was not possible in 3D in thick tissue without sectioning the tissue, thereby necessitating the use of CLSM for this work.

## 4 Discussion

This is the first optical system to demonstrate both absorption and confocal fluorescence contrast. This multimodal system is sensitive to both fluorescence emission and photoacoustic initial pressures from target molecules simultaneously. The complementary contrast is demonstrated here using phantom targets as well as to validate 2D and 3D UV-PARS virtual histology.

UV-PARS demonstrates excellent colocalization with nuclei-staining fluorescent labels in cell cultures and both thick and thin tissues. Cell cultures imaged using epifluorescence and UV-PARS demonstrated excellent colocalization with Li’s ICA of 0.44, clearly defining strong similarities between both these imaging modalities. The minor discrepancy between them can be attributed to resolution differences both laterally and axially. Thin tissue sections were also verified to have strong similarities between UV-PARS and epifluorescence in fresh mouse kidney tissue. In the future work, rather than a 405-nm laser, an LED or mercury lamp could be used as an epifluorescence excitation source, which may provide more homogeneous and speckle-free illumination. Additionally, thick tissue presented difficulties with epifluorescence as the fluorescence emission from multiple cell layers was captured producing a blurred maximum intensity projection of nuclei from several cell layers. UV-PARS was able to optically section individual cell layers of the thick tissue due to its 1.6  μm axial resolution. Fluorescence-based comparisons for thick tissue imaging necessitated the use of CLSM to properly compare and validate cell nuclei images with UV-PARS, the result of which proved to have exemplary colocalization with Li’s ICA average value of 0.445±0.013. Previous work has been able to acquire images in thick tissue but without an alternative microscopy method to validate the UV-PARS images.^26^^,^^27^ There is a great need for a new microscopy methodology to optically section individual cell layers in thick tissues. This imaging methodology is of interest as it decreases the amount of preparation and labor needed to diagnose margin status. Using our microscopy system, 3D virtual H&E staining can be accomplished intraoperatively without further preparation or physical coupling, as needed in H&E staining, microscopy with ultraviolet surface excitation or OR-PAM.^35^^–^^37^

Further work could include imaging endogenous fluorophores including other molecules of interest such as flavin adenine dinucleotide and nicotinamide adenine dinucleotide to measure for metabolic activity to identify cancerous cells.^38^^,^^39^ Additional system improvements will involve imaging larger FOVs and moving toward faster image acquisition. Currently, the presented UV-PARS and CLSM system can produce 0.5  mm×0.5  mm FOV images in around 45 s. However, faster scanning will be needed for practical imaging of larger FOVs. Future work would include the addition of faster laser repetition rates for an increase in acquisition speed potentially suitable for practical intraoperative imaging.

The combined system reported here could augment fluorescence-guided surgeries, where fluorescence from tumor margins may not be well-defined due to variable drug perfusion or staining^40^^–^^42^ and where spot-checking margins with a virtual histological imaging method such as UV-PARS could provide important added specificity. More fundamentally, the combined fluorescence and absorption contrast provided here could find applications for imaging both fluorescent and non-fluorescent absorbing molecules in future cell biology and intravital imaging research.

## 5 Conclusion

UV-PARS microscopy is co-integrated with epifluorescence and CLSM systems to provide both absorption and fluorescence contrast. Camera epifluorescence demonstrated validation of UV-PARS in 2D tissue sections while exposing some of its own shortcomings in thick tissue and the need for imaging capabilities of single cellular layers. Three-dimensional UV-PARS and complimentary CLSM were demonstrated in thick tissues to mark cell nuclei and validate UV-PARS for the first time. CLSM and UV-PARS achieved excellent optical sectioning capabilities of 2.0 and 1.6  μm, respectively. Future applications using real-time imaging could include examples such as multicontrast live-cell imaging, intravital microscopy of vasculature, ${\mathrm{SO}}_{2}$ imaging with complimentary tumor microenvironment and virtual histology applications where the combined absorption and fluorescence contrast would prove efficacious.
